# Prognostic factors and population-based analysis of melanoma with sentinel lymph node biopsy

**DOI:** 10.1038/s41598-021-99950-1

**Published:** 2021-10-15

**Authors:** Ping-Chung Wu, Yu-Ching Chen, Hsiu-Min Chen, Lee-Wei Chen

**Affiliations:** 1 Department of Surgery, Kaohsiung Veterans General Hospital, No. 386, Da-Zhong 1st Rd., Kaohsiung, Taiwan, Republic of China; 2 School of Medicine, National Yang Ming Chiao Tung University, No. 155, Sec. 2, Linong St., Taipei, Taiwan, Republic of China; 3 Department of Medical Education and Research, Kaohsiung Veterans General Hospital, Kaohsiung, Taiwan; 4 Research Center of Medical Informatics, Kaohsiung Veterans General Hospital, No. 386, Da-Zhong 1st Rd., Kaohsiung, Taiwan, Republic of China; 5 Department of Biological Sciences, National Sun Yat-Sen University, Kaohsiung, Taiwan; 6 Institute of Emergency and Critical Care Medicine, National Yang Ming Chiao Tung University, Taipei, Taiwan

**Keywords:** Melanoma, Cancer epidemiology, Surgical oncology, Prognosis

## Abstract

Cutaneous malignant melanoma is a rare but fatal disease in East Asia. Despite its increasing incidence, a general lack of awareness about the disease was noted. This study aims to provide population-based prognostic analysis of melanoma with sentinel lymph node biopsy (SLNB) in Taiwan. We conducted this retrospective cohort study using the data from Taiwan National Health Insurance Research Database during 1997–2013. The study cohort contains 3284 patients. The 5-year survival rates of patients undergoing SLNB and not undergoing SLNB were 45.5% and 33.6%. In multivariate analysis, age ≥ 80 years [adjusted hazard ratio (aHR) = 2.15] and male (aHR = 1.19) were associated with a poorer prognosis, while high social economic status (SES) (aHR = 0.69) and undergoing SLNB (aHR = 0.84) were good prognostic factors. Old age and low SES were associated with lower percentages of patients undergoing SLNB (*P* < 0.001). E-value analysis suggested robustness to unmeasured confounding. In conclusion, undergoing SLNB was associated with a better prognosis. The poor prognosis of old age and low SES may be due to decreased percentages of patients undergoing SLNB. Therefore, we recommend that SLNB should be performed on patients, especially in old age or low SES, who are candidates for SLNB according to current guidelines to achieve maximal survival.

## Introduction

Cutaneous malignant melanoma is a rare but fatal disease in East Asia, with increasing incidence in Taiwan according to Cancer Registry Annual Report published by Ministry of Health and Welfare, Taiwan in 2018^1^. In spite of the increasing incidence of melanoma, a general lack of awareness about the disease and the fact that melanoma remains overlooked and undertreated in Asian populations were noted in recent studies^2,3^. It’s worth mentioning that the two major subtypes in white people are superficial spreading melanoma and nodular melanoma, whereas acral lentiginous melanoma, which is the most common subtype in Asians, is actually rare in the Western countries^4,5^. Therefore, to better understand this disease, statistical data regarding demographic characteristics of melanoma in Asian populations seems to be basic and indispensable. However, studies focusing on this area appears to be limited. To our knowledge, no large-scale, population-based study was performed to provide demographic characteristics of melanoma patients in Taiwan in the past.

Sentinel lymph node biopsy (SLNB) is the most accurate staging tool and offers useful prognostic information for melanoma patients. SLNB has become the standard of care in the treatment of clinically node-negative melanoma. According to current National Comprehensive Cancer Network (NCCN) guideline^6^, SLNB should be discussed with all patients with clinical stage IB or II melanoma. However, controversies of SLNB exist because of the lack of clear evidence regarding the survival advantage SLNB provides^7–10^. One randomized study^11^ in 2014 has been designed to resolved this issue and showed that SLNB leads to an improved survival in node-positive patients, but debate remains as most melanoma patients have negative nodes^12^. In Asia, few studies have been performed to analyze the difference in prognosis between melanoma patients undergoing SLNB or not undergoing SLNB^13,14^.

As a result, this study aims to provide prognostic factors and population-based analysis of melanoma with SLNB in Taiwan.

## Materials and methods

### Data sources

The Taiwan National Health Insurance (NHI) program was initiated in March 1995 and covers approximately 99% of the Taiwanese population. The NHI Research Database (NHIRD), derived from the payment system of the NHI Administration (NHIA) and managed by the National Health Research Institute (NHRI), possesses abundant information regarding nearly all kinds of medical services, such as outpatient visits, inpatient records, and medical illness diagnosis codes according to the International Classification of Diseases, Ninth Revision, Clinical Modification (ICD-9-CM) coding system. Confidentiality is maintained according to the directives of the NHIA in Taiwan. Details on the generation, monitoring and maintenance of the NHIRD are published online by the NHRI. Previous studies have described the high accuracy and validity of ICD-9-CM diagnoses in the NHIRD^15,16^. All data from the NHIRD are anonymous and encrypted to protect participants’ privacy; therefore, no informed consent was required from the study population. Additionally, this study was approved by the Institutional Review Board of Kaohsiung Veterans General Hospital, Kaohsiung, Taiwan (VGHKS15-EM4-01).

### Study design

**Figure 1:**
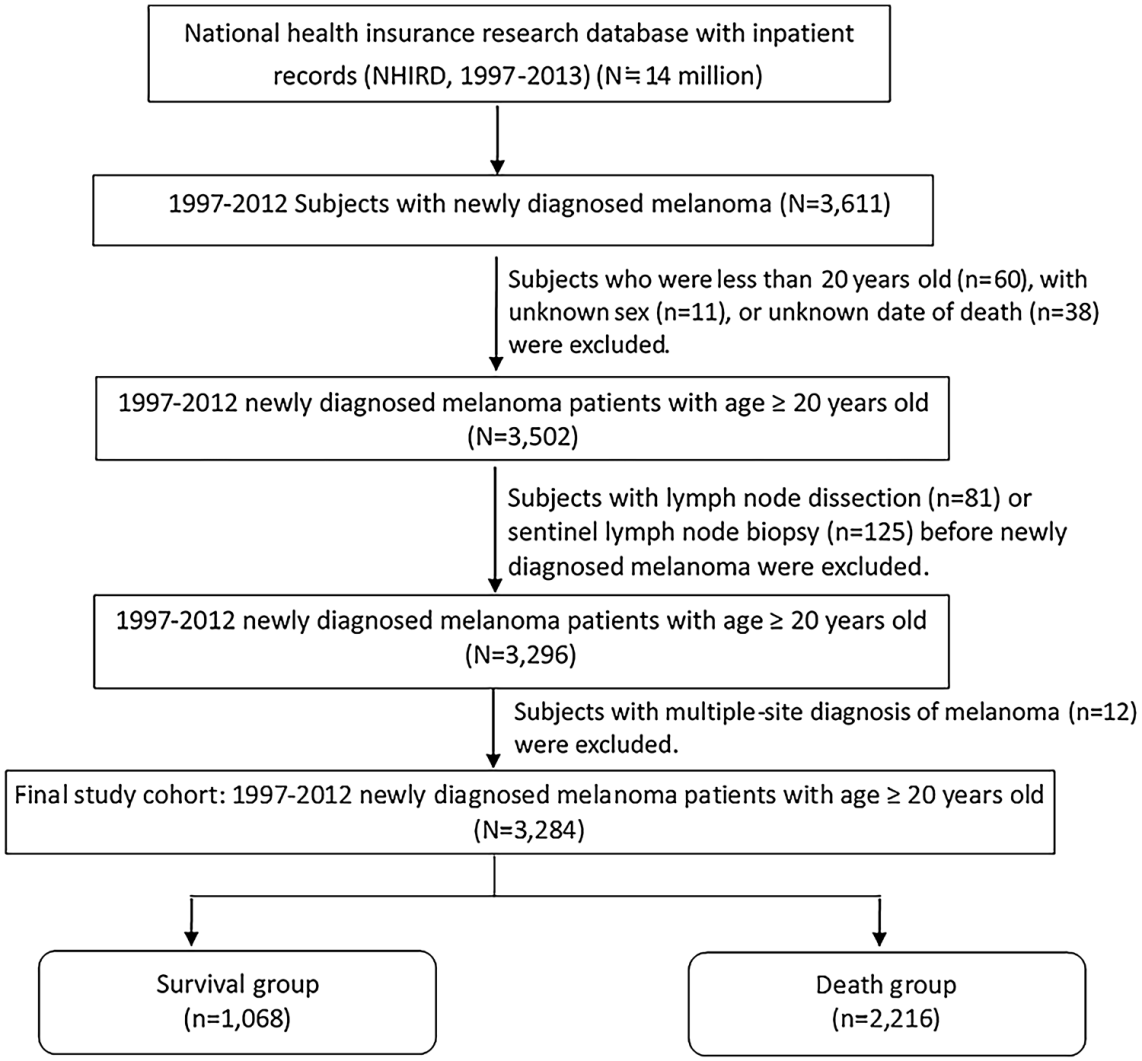
Design and flowchart of patient selection.

We conducted this retrospective cohort study using the data extracted from the inpatient records of NHIRD during 1997–2013, with a total patient number of approximately 14 million. Adults with newly diagnosed malignant melanoma of skin (ICD-9-CM Code 172) were identified from the inpatient claims as the cutaneous malignant melanoma cohort. Exclusion criteria included follow-up time less than 1 year, unknown sex, unknown date of death, age below 20 years, undergoing SLNB or lymph node dissection (LND) before the diagnosis of melanoma, and multiple-site diagnosis of melanoma. The final study cohort contains 3284 patients. Figure 1 shows detailed information on the enrollment process. Overall, 3284 melanoma patients were followed up until death or the end of 2013, with 1068 patients in the survival group and 2216 patients in the death group. According to Cancer Registry Annual Report published by Ministry of Health and Welfare, Taiwan in 2018^1^, the total number of patients diagnosed with melanoma during 1997–2012 was 3057, which was close to the cohort number of this study. The Taiwan Cancer Registry Annual Report was based on the Taiwan Cancer Registry database, which provides complete core information for cancer cases in Taiwan^17,18^.

Demographic data were derived from NHIRD, including age at diagnosis, sex, social economic status (SES) and residential area. SES was classified as non-income (no income), low (income ranges from 1 to 583 US$ per month), middle (income ranges from 584 to 833 US$ per month) and high (income ≥ 834 US$ per month) categories. The baseline comorbidity history for each participant was also determined from the inpatient claims data. Several possibly relevant diseases, according to previous studies^19–22^, including diabetes (ICD-9-CM Code 250), coronary artery disease (ICD-9-CM Codes 410–414), cerebral vascular disease (ICD-9-CM Codes 430–438), heart failure (ICD-9-CM Code 428), Parkinson disease (ICD-9-CM Code 332) and all cancers (ICD-9-CM Codes 140–208), were considered. As for clinical characteristics, primary site of cutaneous melanoma was assessed by ICD-9-CM Codes, with 172.0–172.4 for head and neck, 172.5 for trunk, 172.6 for upper limbs and 172.7 for lower limbs. ICD-9-CM Codes 172.8–172.9 or simply 172 were defined as unspecific group, in addition. Patients undergoing SLNB (procedure codes 4011, 4021, 4023, 4024 and 4029) and LND (procedure codes 403, 4041, 4051, 4053, 4054 and 4059) were also identified.

### Sentinel lymph node biopsy

Current NCCN guideline indicates that SLNB should be discussed with all patients with clinical stage IB or II melanoma^6^. In this study, on account of the lack of consistent guidelines throughout the relatively long time frame, SLNB was performed mainly based on the clinical consideration of clinicians. In Taiwan, generally, the procedure of SLNB included subdermal injection of 1 mL of technetium-99 sulfur colloid and vital dye followed by lymphoscintigraphy and surgical harvest of sentinel lymph nodes^23^. Dissected sentinel lymph nodes were further stained with hematoxylin and eosin or HMB-45.

### Statistical analysis

In this study, survival was analyzed by the Kaplan–Meier method. Univariate analysis of the association between survival and prognostic factors including demographic variables, comorbidities and clinical characteristics was performed using Chi-square test. Multivariate analysis was further performed via the Cox proportional hazard model. As for SLNB, univariate analysis of the association between demographic characteristics as predictive factors and SLNB was also performed using Chi-square test. In addition, subgroup analysis of age, sex, and SES for SLNB by prognosis was further performed using Chi-square test. A *P* value of 0.05 or less was regarded to be statistically significant. SPSS statistical software, version 19.0 for Windows, was used for all data analysis.

### Sensitivity analysis

Due to the limitation of NHIRD, the data of several important clinical characteristics such as Breslow thickness, stage and histologic subtype of melanoma was not analyzed in this study. To assess the robustness of our findings, multiple sensitivity analyses were performed. First, we modified the patient enrollment process by adding another exclusion criterion of follow-up time less than 3 years (see Supplementary Fig. 1 online). The association between SLNB and the end point of 1-year or 3-year overall survival was assessed using crude analysis, multivariable analysis and propensity-score analyses. Second, we explored the potential for unmeasured confounding between SLNB and 1-year or 3-year overall survival by calculating E-values^24^. The E-value is the minimum strength that an unmeasured confounder would need to have to negate the observed association between SLNB and 1-year or 3-year overall survival.

## Results

### Demographic characteristics

Of all 3284 patients, the mean age (± standard deviation) was 65.2 (± 16.1) years. The most common age of diagnosis was between 70 and 79 years. Old age was associated with a significantly increased risk of death (*P* < 0.001). There were 1791 males and 1493 females. The male-to-female ratio was 1.20:1. Male gender was associated with a worse survival (*P* < 0.001) than female gender. As for SES, there were 1485, 711, 666 and 373 patients in middle, low, high and non-income groups, respectively. High SES was associated with a better survival (*P* < 0.001) than other groups. Most of the patients resided in northern Taiwan, while there’s no significant association between residential area and survival. Details of the demographic characteristics of the melanoma patients are described in Table 1.

**Table 1:** Demographic characteristics, comorbidity and clinical characteristics of first-event melanoma patients in survival and death groups (*n* = 3284).

Variables	Total	Survival (*n* = 1,068)	Death (*n* = 2,216)	*p* Value^a^
**Demographics**				
Age, years, mean ± SD (%)	65.2 ± 16.1	59.0 ± 16.1	68.2 ± 15.2	** < 0.001**
< 50	584 (17.8)	299 (51.2)	285 (48.8)	** < 0.001**
50–59	495 (15.1)	219 (44.2)	276 (55.8)	
60–69	670 (20.4)	228 (34.0)	442 (66.0)	
70–79	903 (27.5)	218 (24.1)	685 (75.9)	
≥ 80	632 (19.2)	104 (16.5)	528 (83.5)	
Sex (%)				** < 0.001**
Female	1493 (45.5)	565 (37.8)	928 (62.2)	
Male	1791 (54.5)	503 (28.1)	1288 (71.9)	
Social economic status (%)^bc^				** < 0.001**
Non-income	373 (11.5)	80 (21.4)	293 (78.6)	
Low	711 (22.0)	205 (28.8)	506 (71.2)	
Middle	1485 (45.9)	432 (29.1)	1053 (70.9)	
High	666 (20.6)	333 (50.0)	333 (50.0)	
Residential area (%)^d^				0.130
Northern	1304 (40.7)	450 (34.5)	854 (65.5)	
Central	814 (25.4)	251 (30.8)	563 (69.2)	
Southern	952 (29.7)	301 (31.6)	651 (68.4)	
Other	137 (4.3)	37 (27.0)	100 (73.0)	
**Comorbidity**				
Diabetes (%)				** < 0.001**
No	2725 (83.0)	926 (34.0)	1799 (66.0)	
Yes	559 (17.0)	142 (25.4)	417 (74.6)	
Coronary artery disease (%)				** < 0.001**
No	2262 (68.9)	787 (34.8)	1475 (65.2)	
Yes	1022 (31.1)	281 (27.5)	741 (72.5)	
Cerebral vascular disease (%)				** < 0.001**
No	2907 (88.5)	1004 (34.5)	1903 (65.5)	
Yes	377 (11.5)	64 (17.0)	313 (83.0)	
Heart failure (%)				** < 0.001**
No	3087 (94.0)	1036 (33.6)	2051 (66.4)	
Yes	197 (6.0)	32 (16.2)	165 (83.8)	
Parkinson disease (%)				0.240
No	3225 (98.2)	1053 (32.7)	2172 (67.3)	
Yes	59 (1.8)	15 (25.4)	44 (74.6)	
Malignancy (%)				** < 0.001**
No	2041 (62.1)	860 (42.1)	1181 (57.9)	
Yes	1243 (37.9)	208 (16.7)	1035 (83.3)	
**Melanoma site (%)**				** < 0.001**
Head and neck	488 (14.9)	141 (28.9)	347 (71.1)	
Trunk	310 (9.4)	107 (34.5)	203 (65.5)	
Upper limbs	271 (8.3)	123 (45.4)	148 (54.6)	
Lower limbs	1,622 (49.4)	585 (36.1)	1,037 (63.9)	
Unspecific	593 (18.1)	112 (18.9)	481 (81.1)	
**Sentinel lymph node biopsy (%)**				** < 0.001**
No	2,351 (71.6)	667 (28.4)	1,684 (71.6)	
Yes	933 (28.4)	401 (43.0)	532 (57.0)	
**Lymph node dissection (%)**				**0.002**
No	2,813 (85.7)	945 (33.6)	1,868 (66.4)	
Yes	471 (14.3)	123 (26.1)	3483.9)	

^a^Using Chi-square test. Bold type indicates statistical significantly (*P* < 0.05).

^b^Social economic status was classified as non-income (no income), low (income ranges from 1 to 583 US$ per month), middle (income ranges from 584 to 833 US$ per month) and high (income ≥ 834 US$ per month) categories.

^c^49 patients were unknown.

^d^77 patients were unknown.

### Comorbidity

Among all the comorbidities considered, the most common comorbidity in these 3284 melanoma patients was malignancy (37.9%), followed by coronary artery disease (31.1%) and diabetes (17.0%). On the other hand, the least common comorbidity was Parkinson disease (1.8%), followed by heart failure (6.0%) and cerebral vascular disease (11.5%). In the univariate analysis, the diagnosis of malignancy, coronary artery disease, diabetes, cerebral vascular disease and heart failure were associated with an increased risk of death (*P* < 0.001). Details of the prevalence of comorbidity in survival and death groups are also shown in Table 1.

### Clinical characteristics

According to the ICD-9-CM Codes, the most commonly diagnosed site was on lower limbs (49.4%), followed by unspecific sites (18.1%), head and neck (14.9%), trunk (9.4%) and upper limbs (8.3%). Of the 3284 cases, we identified 933 (28.4%) patients undergoing SLNB, which was associated with a better prognosis (*P* < 0.001). In addition, 471 (14.3%) patients underwent LND, which was associated with an increased risk of death (*P* = 0.002). Detailed clinical characteristics of melanoma patients in survival and death groups are also shown in Table 1.

### Overall survival

**Figure 2:**
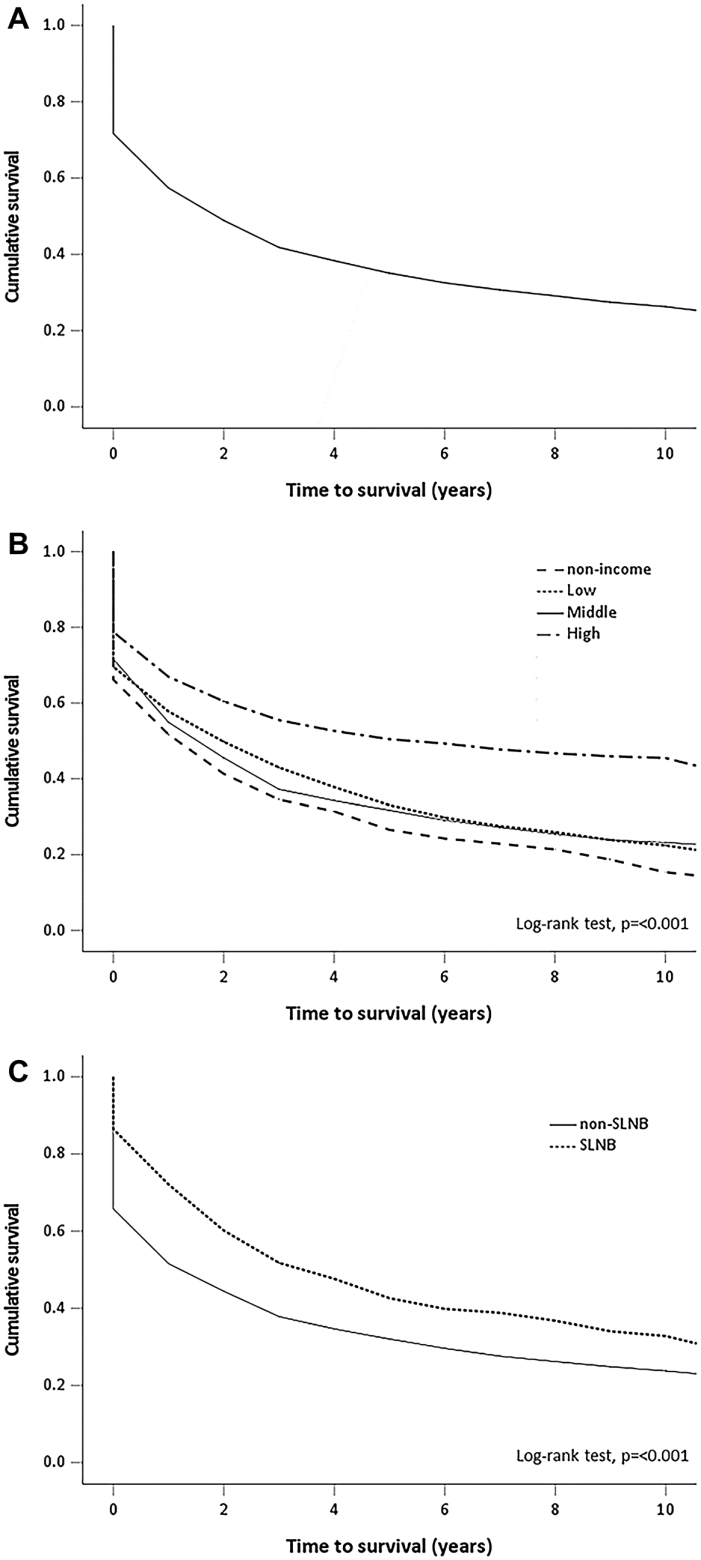
Overall survival of patients with malignant melanoma in Taiwan. (**A**) Cumulative proportion of the 3284 patients expected to survive. (**B**) Patients in different categories of social economic status. (**C**) Patients undergoing sentinel lymph node biopsy or not.

The overall survival curve for these 3284 melanoma patients made by the Kaplan–Meier method is shown in Fig. 2. The 1-year, 3-year, 5-year and 10-year overall survival rate were 71.7%, 47.3%, 37.0% and 26.3%, respectively. High SES and undergoing SLNB were associated with a significantly better prognosis. The overall survival curves of patients with different SES and patients undergoing SLNB or not are shown in Fig. 2. The 5-year survival rates of patients with high, middle, low and non-income SES were 51.4%, 33.3%, 36.1% and 29.6%, respectively. The 5-year survival rates of patients undergoing SLNB and not undergoing SLNB were 45.5% and 33.6%.

### Multivariate analysis

In the univariate analysis, age, sex, SES, comorbidity, site of melanoma, undergoing SLNB and LND were significantly associated with prognosis. To avoid confounding and to better reflect independent prognostic factors, Table 2 provides the multivariate analysis of these factors. Patients aged ≥ 80 years [adjusted hazard ratio (aHR) = 2.15], between 70 and 79 years of age (aHR = 1.63), between 60 and 69 years of age (aHR = 1.38) or between 50 and 59 years of age (aHR = 1.22) had a worse prognosis than patients aged less than 50 years. Male gender was associated with a worse survival (aHR = 1.19) than female gender. The aHRs for low, middle and high SES were 0.84 (95% CI 0.72–0.97), 0.90 (95% CI 0.78–1.02) and 0.69 (95% CI 0.58–0.83), respectively, compared with non-income group. Comorbidities including diabetes, coronary artery disease and heart failure were significantly poor prognostic factors in univariate analysis, but was not significant in multivariate analysis. Cerebral vascular disease (aHR = 1.22, *P* = 0.002) and malignancy (aHR = 2.13, *P* < 0.001) remained significant poor prognostic factors in multivariate analysis. Undergoing SLNB (aHR = 0.84, *P* = 0.001) and LND (aHR = 1.15, *P* = 0.022) were also significantly prognostic factors in multivariate analysis.

**Table 2:** Demographic and clinical characteristics of patients in the mortality by Cox-proportional hazard regression analysis with stepwise model (*n* = 3284).

Variables	aHR^b^	95% C.I.^b^	*p* Value^a^
**Demographics**			
Age, years (ref. = < 50)			
50–59	1.22	1.03–1.45	**0.021**
60–69	1.38	1.19–1.62	** < 0.001**
70–79	1.63	1.40–1.89	** < 0.001**
≥ 80	2.15	1.83–2.54	** < 0.001**
**Sex (ref. = female)**			
Male	1.19	1.09–1.30	** < 0.001**
**Social economic status (ref. = Non-income)**^**c**^			
Low	0.84	0.72–0.97	**0.020**
Middle	0.90	0.78–1.02	0.105
High	0.69	0.58–0.83	** < 0.001**
**Melanoma site (ref. = Head and neck)**			
Trunk	1.10	0.92–1.32	0.277
Upper limb	0.92	0.76–1.12	0.426
Lower limb	0.97	0.85–1.10	0.636
Unspecific	1.27	1.10–1.46	**0.001**
**Comorbidity (ref. = No)**			
Cerebral vascular disease	1.22	1.07–1.38	**0.002**
Malignancy	2.13	1.95–2.33	** < 0.001**
**Clinical surgery (ref. = No)**			
Sentinel lymph node biopsy	0.84	0.76–0.93	**0.001**
Lymph node dissection	1.15	1.02–1.30	**0.022**

^a^Using Cox proportional hazard model. Bold type indicates statistical significantly (*P* < 0.05).

^b^Abbreviations: *aHR* adjusted hazard ratio, *C.I.* confidence interval.

^c^Social economic status was classified as non-income (no income), low (income ranges from 1 to 583 US$ per month), middle (income ranges from 584 to 833 US$ per month) and high (income ≥ 834 US$ per month) categories.

### Sentinel lymph node biopsy

Of all 3284 melanoma patients, 933 (28.4%) cases underwent SLNB whereas 2351 (71.6%) cases didn’t undergo SLNB. Sex was not associated with the percentage of patients undergoing SLNB, while old age and low SES were associated with a lower percentage of patients undergoing SLNB (*P* < 0.001). Details of percentage of patients undergoing SLNB in different age, sex and SES categories are shown in Table 3. To further investigate the relationship between SLNB and prognosis, we conducted the subgroup analysis of age, sex and SES for SLNB by prognosis (Table 4). Among patients in different age, sex and SES subgroups, SLNB was associated with a significantly better prognosis, except for patients aged less than 50 years (*P* = 0.105) and the high SES subgroup (*P* = 0.175).

**Table 3:** Percentage of patients undergoing SLNB in different age, sex, SES categories (*n* = 3284).

Variables	Sentinel lymph node biopsy		*p* Value^a^
	No (*n* = 2351)	Yes (*n* = 933)	
**Age, years (%)**			** < 0.001**
< 50	370 (63.4)	214 (36.6)	
50–59	304 (61.4)	191 (38.6)	
60–69	460 (68.7)	210 (31.3)	
70–79	679 (75.2)	224 (24.8)	
≥ 80	538 (85.1)	94 (14.9)	
**Sex (%)**			0.826
Female	1066 (71.4)	427 (28.6)	
Male	285 (71.7)	506 (28.3)	
**Social economic status (%)**^**bc**^			** < 0.001**
Non-income	286 (76.7)	87 (23.3)	
Low	545 (76.7)	166 (23.3)	
Middle	1066 (71.8)	419 (28.2)	
High	411 (61.7)	255 (38.3)	

^a^Using Chi-square test. Bold type indicates statistical significantly (*P* < 0.05).

^b^Social economic status was classified as non-income (no income), low (income ranges from 1 ot 583 US$ per month), middle (income ranges from 584 to 833 US$ per month) and high (income ≥ 834 US$ per month) categories.

^c^49 patients were unknown.

**Table 4:** Subgroup analysis of age, sex and social economic status for SLNB by prognosis (*n* = 3284).

Variables	Survival (*n* = 1068)	Death (*n* = 2216)	*p* Value^a^
**Age**			
< 50 years (%)			0.105
Non-SLNB	180 (48.6)	190 (51.4)	
SLNB	119 (55.6)	95 (44.4)	
50–59 years (%)			**0.012**
Non-SLNB	121 (39.8)	183 (60.2)	
SLNB	98 (51.3)	93 (48.7)	
60–69 years (%)			**0.043**
Non-SLNB	145 (31.5)	315 (68.5)	
SLNB	83 (39.5)	127 (60.5)	
70–79 years (%)			**0.001**
Non-SLNB	146 (21.5)	533 (78.5)	
SLNB	72 (32.1)	152 (67.9)	
≥ 80 years (%)			** < 0.001**
Non-SLNB	75 (13.9)	463 (86.1)	
SLNB	29 (30.9)	65 (69.1)	
**Sex**			
Female (%)			** < 0.001**
Non-SLNB	341 (32.0)	725 (68.0)	
SLNB	224 (52.5)	203 (47.5)	
Male (%)			** < 0.001**
Non-SLNB	326 (25.4)	959 (74.6)	
SLNB	177 (35.0)	329 (65.0)	
**Social economic status**^bc^			
Non-income (%)			** < 0.001**
Non-SLNB	48 (16.8)	238 (83.2)	
SLNB	32 (36.8)	55 (63.2)	
Low (%)			**0.001**
Non-SLNB	140 (25.7)	405 (74.3)	
SLNB	65 (39.2)	101 (60.8)	
Middle (%)			** < 0.001**
Non-SLNB	270 (25.3)	796 (74.7)	
SLNB	162 (38.7)	257 (61.3)	
High (%)			0.175
Non-SLNB	197 (47.9)	214 (52.1)	
SLNB	136 (53.3)	119 (46.7)	

^a^Using Chi-square test. Bold type indicates statistical significantly (*P* < 0.05).

^b^Social economic status was classified as non-income (no income), low (income ranges from 1 to 583 US$ per month), middle (income ranges from 584 to 833 US$ per month) and high (income ≥ 834 US$ per month) categories.

^c^49 patients were unknown.

### Sensitivity analysis

There are 2823 patients in our sensitivity analysis. The 1-year and 3-year overall survival rate were 70.7% and 47.5%, respectively (see Supplementary Fig. 1 online). The association between SLNB and 1-year or 3-year overall survival in the crude analysis, multivariable analysis and propensity-score analyses was demonstrated in Table 5, which showed consistently better survival in the SLNB group. Moreover, the results of E-value analysis were shown in Table 6. In propensity-score analysis with matching, undergoing SLNB was associated with a better 1-year overall survival that had a hazard ratio of 0.40 (95% CI 0.31–0.50). The E-value for this was 3.16, meaning that residual confounding could explain the observed association if there exists an unmeasured covariate having a relative risk association at least as large as 3.16 with both 1-year overall survival and with undergoing SLNB. The baseline characteristics before and after propensity score matching can be seen as Supplementary Table 1 online, while univariate analysis with survival outcomes can be found as Supplementary Table 2 online.

**Table 5:** Association between SLNB and the End Point of 1-year or 3-year Overall Survival in the Crude Analysis, Multivariable Analysis and Propensity-Score Analyses.

Analysis	1-Year overall survival	3-Year overall survival
**No. of events/no. of patients at risk (%)**		
No SLNB	717/2044	1156/2044
SLNB	109/779	326/779
Crude analysis—hazard ratio (95% CI)	0.34 (0.28, 0.42)	0.59 (0.52, 0.67)
Multivariable analysis—hazard ratio (95% CI)*	0.45 (0.36, 0.57)	0.71 (0.62, 0.82)
**Propensity-score analyses—hazard ratio (95% CI)**		
With inverse probability weighting^†^	0.41 (0.30, 0.54)	0.81 (0.65, 0.99)
With matching^‡^	0.40 (0.31, 0.50)	0.70 (0.60, 0.81)

*Shown is the hazard ratio from the multivariable Cox proportional-hazards model, with adjustment for age, sex, social economic status, cancer sites, lymph node dissection, diabetes, hypertension, coronary artery disease, stroke, congestive heart failure, cirrhosis, Parkinson disease, COPD, malignancy. The analysis included all 2823 patients.

^†^Shown is the primary analysis with a hazard ratio from the multivariable Cox proportional-hazards model with covariates with inverse probability weighting according to the propensity score. The analysis included 2823 patients.

^‡^Shown is the hazard ratio from a multivariable Cox proportional-hazards model with covariates with matching according to the propensity score. The analysis included 2049 patients (1366 without SLNB and 683 undergoing SLNB).

**Table 6:** Evaluation for unmeasured confounding between SLNB groups by E-value analysis.

Analysis	1-Year overall survival	3-Year overall survival
Crude analysis—E value	3.60	2.24
Multivariable analysis—E value	2.86	1.85
**Propensity-score analyses**		
With inverse probability weighting—E value	3.09	1.58
With matching—E value	3.16	1.88

The larger the E-value, the lower the probability that an unmeasured confounder was to explain the entirety of the treatment effect.

## Discussion

Old age was associated with a worse prognosis in previous studies in Taiwan^4^ and Western countries^25–27^. Age has been proved to be a very strong and independent predictor of survival outcome after accounting for all the dominant prognostic factors^27^. One reason that old age may be associated with a poor prognosis is that melanomas in older patients were more frequently late diagnosed^26^, which may be related to less attention to changes on their skin, low awareness of the early signs and symptoms of melanoma, increase of seborrheic keratoses (with which melanoma can be easily confused) or development of a higher proportion of melanomas in hard-to-see anatomical sites^28^. Another possible reason for old age being a poor prognostic factor is that melanomas in older patients were generally thicker, more likely to be ulcerated and with higher mitotic rates, which are thought to be prognostically unfavorable features^26,27^. One review article in 2018 has discussed the relationship between age and sentinel lymph node status^29^ and indicated decreased sentinel lymph node positivity but increased mortality with age. This may be due to the decreased possibility of senescent melanocytes reaching sentinel nodes and the inability of local immune system to control melanocytes when they reach sentinel nodes. In this study, old age was associated with a significantly increased risk of death (*P* < 0.001), which was consistent with former studies^4,25–27^.

According to cancer registries in Taiwan, the age‑adjusted incidence rates for invasive melanoma in 2018 were 0.77/100,000 for males and 0.64/100,000 for females, with the age‑adjusted male‑to‑female ratio of 1.20:1^1^. One Taiwanese study of 181 melanoma cases in 2004 reported a male‑to‑female ratio of 0.88:1^4^, while another study of 56 cases in 2019 reported a male‑to‑female ratio of 3.67:1^30^. In our study, the male-to-female ratio of 3284 melanoma cases was 1.20:1, which was between the data of the two above-mentioned studies and was closer to the data in 2018 cancer registries in Taiwan. This ratio difference may be result from the larger case number and the population-based case source in our study. Many studies have demonstrated that female gender confers a better prognosis than male gender^31,32^, which was also shown in this study. The underlying protective effect of female gender is not well understood. Possible explanations include a greater incidence of unfavorable primary tumor characteristics^32^ and an older age at which melanoma were presented with in male patients^31,33^.

Although few comorbidities were thought to be closely related to melanoma, previous studies have suggested that cardiovascular disease was a significant risk factor for melanoma development while diabetes was a non-significant protective factor for melanoma development^19,20^. In addition, it’s also reported that there appears to be an association between melanoma and Parkinson disease^21^. In this study, these possibly relevant comorbidities were taken as prognostic factors and analyzed using a total of 3284 melanoma cases. Multivariate analysis showed that cerebral vascular disease (aHR = 1.22, *P* = 0.002) and malignancy (aHR = 2.13, *P* < 0.001) were significantly poor prognostic factors. However, the relationships between melanoma and these diseases and the clinical significance of our results remained unclear due to limited relevant studies at present. Further studies regarding this topic would be needed to unveil more details.

Whether anatomic site of melanoma is a significant predictor of survival remains controversial due to inconsistent statistical data among previous studies. According to a review article in 2009^34^, many studies have suggested that extremity lesions offer a better prognosis than truncal lesions, while other studies have reported a completely opposite result. There’re still other studies which have failed to demonstrate the prognostic significance of anatomic location, particularly when the studies controlled for thickness. The discrepancy between these studies has yet to be explained, but may be possibly caused by diverse sample sizes, inconsistent study design and different confounding factors considered in previous studies. The fact that the most common anatomic sites differ in different melanoma subtypes, and that the prevalence of each melanoma subtype differs in western and eastern countries, may also contribute to this discrepancy. In this study, anatomic site of cutaneous melanoma, identified using ICD-9-CM Codes, was also analyzed as a prognostic factor. Our data suggested that the most commonly diagnosed site was on lower limbs (49.4%), followed by unspecific sites (18.1%), head and neck (14.9%), trunk (9.4%) and upper limbs (8.3%). In multivariate analysis, the only significant prognostic factor was unspecific site, with an aHR of 1.27 (95% CI 1.10–1.46, *P* = 0.001), compared with head and neck.

Issues surrounding health inequality have gained increasing attention over the past decades. The association between SES and melanoma was discussed in many Western studies previously^35–39^, yet relevant studies among Asian populations were rare. Several Western studies have demonstrated that melanoma is more common in high SES than in low SES populations^35–38^. Potential explanations for this finding are the more frequent exposure to recreational UV radiation, typically intermittent and high-intensity, in high SES individuals and the possible underreporting of melanoma in low SES individuals^35–37,40^. In contrast, lower SES was shown to be associated with a worse survival in previous studies, which may be a consequence of the relative lack of awareness of the severity of melanoma, delayed diagnosis and less management received after diagnosis^35,41^. Another possible reason may be the fact that acral lentiginous melanoma, which was of a worse prognosis compared to other subtypes, seemed to be associated with farmers, who were generally categorized as low SES group^4,42^. In our study, there were 1485, 711, 666 and 373 patients in middle, low, high and non-income groups, respectively. High SES was associated with a better survival (*P* < 0.001) than other groups in both univariate and multivariate analysis, which was compatible with former studies. Our finding indicated that similar disadvantages including lack of awareness of the disease or lack of access to medical resources among low SES populations may exist in Taiwan.

In the past, LND was usually recommended for patients with positive SLNB results. However, the two recent randomized controlled trials, German Dermatologic Cooperative Oncology Group-Selective Lymphadenectomy Trial (DeCOG-SLT)^43^ and Multicenter Selective Lymphadenectomy Trial II (MSLT-II)^44^, have shown that immediate LND provides no survival benefits. In DeCOG-SLT, there was no significant difference in 3-year distant-metastases-free survival, 3-year overall survival, 3-year recurrence-free survival and regional recurrence rate between patients treated with LND or nodal observation following a positive SLNB. In MSLT-II, no significant difference in melanoma-specific survival was seen between the LND arm and the observation arm. Therefore, LND is likely to be performed with diminishing frequency. In our study, undergoing LND was analyzed as a prognostic factor in melanoma cohort, and was associated with an increased risk of death in univariate (*P* = 0.002) and multivariate analysis (aHR = 1.15, *P* = 0.022). We attributed this result to the fact that patients who received LND were generally in advanced stage of disease and thus had a worse prognosis.

As the most effective method of lymphatic mapping and regional lymph node staging currently, SLNB provides useful prognostic information for clinicians to make ongoing treatment plan^45^. A meta-analysis conducted in 2016 reported that the melanoma-specific survival of patients undergoing SLNB plus wide local excision is better than that of patients undergoing wide local excision alone^46^. To date, the only randomized controlled trial evaluating SLNB is the Multicenter Selective Lymphadenectomy Trial (MSLT-1)^11^, which showed a significantly improved disease-free survival in the biopsy group compared to the observation group, among melanoma patients with lesions ≥ 1.2 mm in thickness. However, controversies exist due to the lack of clear evidence regarding the survival advantage SLNB provides^7–10^. In MSLT-1, no significant treatment-related difference in the 10-year melanoma-specific survival rate was seen in the overall study population. Other concerns included the cost to health system and the risk of complications such as lymphedema^10,47,48^. Despite all the controversies, SLNB for accurate staging has become the standard work-up for patients with intermediate-thickness or thick primary melanomas according to a number of relatively consistent guidelines^49–51^.

Histologic subtype of melanoma has been shown to be an independent prognostic factor in several studies^52,53^. It’s worth mentioning that the two major subtypes in white people are superficial spreading melanoma and nodular melanoma, whereas acral lentiginous melanoma, which is the most common subtype in Asians, is actually rare in the Western countries^4,5^. Therefore, studies evaluating the survival benefit of SLNB among Asian populations seem to be important. However, few studies regarding this area have been conducted so far. A China study comprising 47,351 patients reported that patients who underwent SLNB had significantly longer 5-year rates for overall survival and melanoma-specific survival compared with patients who did not undergo SLNB^54^, and another Taiwan study comprising 209 cases had similar result^14^. In our study of 3284 melanoma patients, undergoing SLNB was a statistically significant good prognostic factor in both univariate and multivariate analysis. In addition, old age and low SES were associated with a decreased percentage of patients undergoing SLNB. This may be due to clinical consideration that older patients are likely to have a higher rate of comorbidities contraindicating SLNB^55^. This may be also due to the lack of willingness or access to medical resources in old age and low SES patients. For example, patients with low SES who are required to work a lot simply to maintain their daily expenditures may prefer conservative management rather than undergoing further survey such as SLNB. Moreover, in subgroup analysis of age, sex and SES for SLNB by prognosis, SLNB cohorts in each subgroup had a better prognosis compared to non-SLNB cohorts. The association between undergoing SLNB and a better prognosis was observed consistently in our statistic data. As a result, we recommend that SLNB should be performed on patients, especially in old age or low SES, who are candidates for SLNB according to current guidelines to achieve maximal survival.

However, this study still has a number of limitations. First, SLNB was not randomly assigned but was examined retrospectively in this study. Second, the data of several important clinical characteristics such as Breslow thickness, stage and histologic subtype of melanoma was unavailable from NHIRD, and thus we could not adjust for these important factors in our analysis. Third, outcomes were evaluated using overall survival rather than melanoma-specific survival due to the limitation of NHIRD. Based on the aforementioned reasons, the result of this study should be interpreted with caution.

To address the potential bias caused by missing several important prognostic factors in this study, we further performed propensity score-based sensitivity analysis and E-value sensitivity analysis. The results of crude analysis, multivariable analysis and propensity-score analysis showed consistently better survival in the SLNB group. In E-value sensitivity analysis, potential implications of unmeasured confounders were quantified and we found that an unmeasured confounder was unlikely to explain the entirety of the treatment effect. Therefore, the robustness of our finding that undergoing SLNB was associated with a better prognosis was ensured by sensitivity analyses.

## Conclusions

Undergoing SLNB was associated with a better prognosis. The poor prognosis of old age and low SES may be due to decreased percentages of patients undergoing SLNB. Therefore, we recommend that SLNB should be performed on patients, especially in old age or low SES, who are candidates for SLNB according to current guidelines to achieve maximal survival. Further studies are needed to provide better evidential support for the survival benefit of SLNB.

## Supplementary Information

Supplementary Information.
