# Supplementary material for: Prognostic factors and population-based analysis of melanoma with sentinel lymph node biopsy
Source: Sci Rep. 2021 Oct 15;11:20524. doi: 10.1038/s41598-021-99950-1 (PMC8521595; doi:10.1038/s41598-021-99950-1)
Supplement: Supplementary file 1 — Supplementary Information. [file 41598_2021_99950_MOESM1_ESM.pdf]

Supplementary Figure 1. Flowchart of patient selection in sensitivity analysis

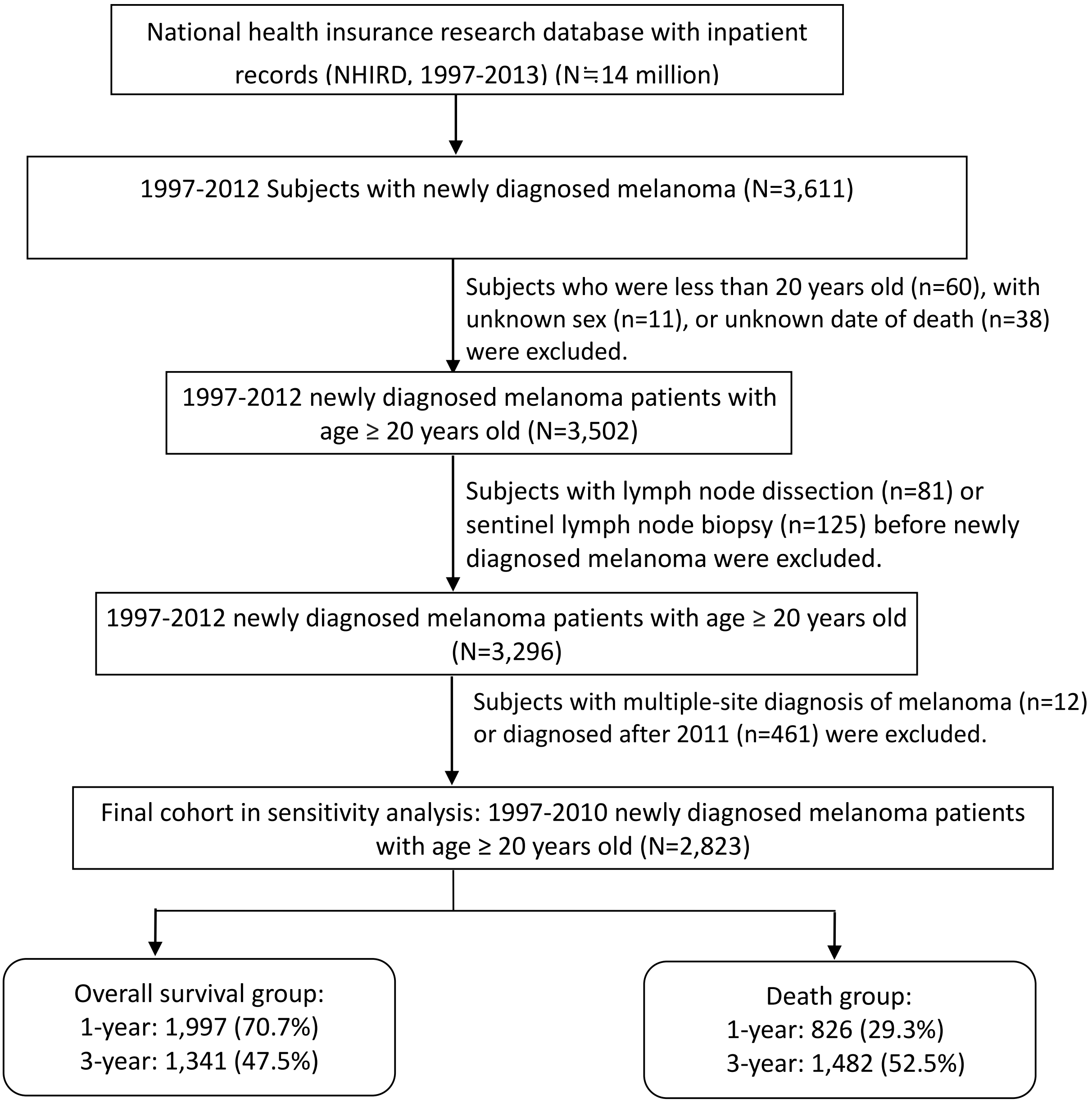

Supplementary Table 1. Baseline characteristics before and after Propensity Score Matching

	Unmatched Patients (N=2823)			Propensity-Score–Matched Patients (N=2049)		
Characteristics	No SLNB (n=2044)	SLNB (n=779)	SMD	No SLNB (n=1366)	SLNB (n=683)	SMD
Age, years, mean±SD	66.4 ± 16.2	61.0 ± 15.7	0.3411	63.5 ± 16.0	62.7 ± 15.4	0.0511
Age group, %						
< 50	332 (16.2)	183 (23.5)	0.1824	261 (19.1)	134 (19.6)	0.0130
50-59	268 (13.1)	154 (19.8)	0.1803	225 (16.5)	121 (17.7)	0.0331
60-69	405 (19.8)	173 (22.2)	0.0588	315 (23.1)	161 (23.6)	0.0121
70-79	590 (28.9)	199 (25.5)	0.0746	354 (25.9)	197 (28.8)	0.0657
≥ 80	449 (22.0)	70 (9.0)	0.3648	211 (15.4)	70 (10.2)	0.1558
Sex, %			0.0181			0.0516
Female	929 (45.5)	353 (45.3)		621 (45.5)	293 (42.9)	
Male	1115 (54.5)	426 (54.7)		745 (54.5)	390 (57.1)	
Social economic status, % [a]						
Non-income	260 (12.9)	77 (9.9)	0.0954	135 (9.9)	77 (11.3)	0.0452
Low	480 (23.9)	145 (18.7)	0.1281	293 (21.4)	138 (20.2)	0.0307
Middle	930 (46.3)	357 (45.9)	0.0069	663 (48.5)	326 (47.7)	0.0161
High	339 (16.9)	198 (25.5)	0.2119	275 (20.1)	142 (20.8)	0.0163
Melanoma site, %						
Head and neck	385 (18.8)	39 (5.0)	0.4368	200 (14.6)	38 (5.6)	0.3047
Trunk	197 (9.6)	71 (9.1)	0.0180	128 (9.4)	69 (10.1)	0.0247
Upper limb	146 (7.1)	82 (10.5)	0.1194	100 (7.3)	78 (11.4)	0.1410
Lower limb	841 (41.1)	533 (68.4)	0.5698	602 (44.1)	452 (66.2)	0.4559
Unspecific	475 (23.2)	54 (6.9)	0.4679	336 (24.6)	46 (6.7)	0.5070
Lymph node dissection, %	292 (14.3)	122 (15.7)	0.0386	219 (16)	102 (14.9)	0.0304
Diabetes, %	356 (17.4)	92 (11.8)	0.1592	179 (13.1)	90 (13.2)	0.0022
Hypertension, %	576 (28.2)	185 (23.7)	0.1012	346 (25.3)	172 (25.2)	0.0034
Coronary artery disease, %	666 (32.6)	182 (23.4)	0.2065	354 (25.9)	175 (25.6)	0.0067
Stroke, %	264 (12.9)	51 (6.5)	0.2161	117 (8.6)	51 (7.5)	0.0404
Congestive heart failure, %	147 (7.2)	17 (2.2)	0.2387	37 (2.7)	17 (2.5)	0.0138
Chronic kidney disease, %	112 (5.5)	23 (3.0)	0.1260	52 (3.8)	23 (3.4)	0.0236
Cirrhosis, %	161 (7.9)	41 (5.3)	0.1056	92 (6.7)	39 (5.7)	0.0424
Parkinson disease, %	41 (2.0)	6 (0.8)	0.1058	13 (1)	6 (0.9)	0.0077
COPD, %	125 (6.1)	15 (1.9)	0.2145	29 (2.1)	15 (2.2)	0.0050
Malignancy, %	292 (14.3)	122 (15.7)	0.0711	498 (36.5)	243 (35.6)	0.0183

a. Social economic status was classified as non-income (no income), low (income ranges from 1-583 US$ per month), middle (income ranges from 584-833 US$ per month) and high (income ≥ 834 US$ per month) categories with 49 patients unknown.; SLNB, sentinel lymph node biopsy; COPD, chronic obstructive pulmonary disease; SMD, a standardized mean difference of 0.1 or less indicates a negligible difference.

Supplementary Table 2. Univariate analysis with survival outcomes

	1-year overall survival		3-year overall survival	
**Characteristics**	**HR (95% CI)**	**P value**	**HR (95% CI)**	**P value**
**Sentinel lymph node biopsy**	**0.38 (0.31, 0.48)**	**<0.0001**	**0.69 (0.60, 0.79)**	**<0.0001**
Age group				
< 50	1.0			
50-59	0.95 (0.70, 1.29)	0.7400	1.17 (0.93, 1.46)	0.1710
60-69	0.98 (0.74, 1.29)	0.8661	1.30 (1.06, 1.59)	0.0127
70-79	1.09 (0.83, 1.42)	0.5366	1.39 (1.15, 1.70)	0.0009
≥ 80	2.11 (1.60, 2.76)	<0.0001	2.29 (1.85, 2.83)	<0.0001
Male	1.25 (1.05, 1.48)	0.0131	1.34 (1.18, 1.51)	<0.0001
Social economic status [a]				
Non-income	1.0		1.0	
Low	1.05 (0.77, 1.43)	0.7645	0.92 (0.73, 1.15)	0.4580
Middle	1.00 (0.75, 1.32)	0.9768	1.05 (0.86, 1.29)	0.6164
High	0.70 (0.50, 0.98)	0.0385	0.68 (0.54, 0.87)	
Melanoma site				0.0019
Head and neck	1.0		1.0	
Trunk	1.23 (0.86, 1.76)	0.2631	1.25 (0.96, 1.63)	0.1020
Upper limb	0.71 (0.46, 1.08)	0.1098	0.75 (0.55, 1.02)	0.0649
Lower limb	0.73 (0.55, 0.98)	0.0339	0.97 (0.79, 1.19)	0.7464
Unspecific	2.28 (1.70, 3.05)	<0.0001	1.87 (1.49, 2.34)	<0.0001
Lymph node dissection	0.54 (0.41, 0.71)	<0.0001	0.99 (0.84, 1.17)	0.8848
Diabetes	1.14 (0.90, 1.46)	0.2766	1.10 (0.92, 1.31)	0.3134
Hypertension	1.03 (0.84, 1.25)	0.7959	0.96 (0.83, 1.11)	0.5764
Coronary artery disease	0.98 (0.81, 1.19)	0.8294	0.97 (0.84, 1.12)	0.7001
Stroke	1.61 (1.23, 2.10)	0.0004	1.62 (1.33, 1.97)	<0.0001
Congestive heart failure	1.12 (0.67, 1.87)	0.6600	1.08 (0.74, 1.58)	0.6740
Chronic kidney disease	1.14 (0.74, 1.76)	0.5623	1.18 (0.86, 1.61)	0.3060
Cirrhosis	1.42 (1.05, 1.92)	0.0249	1.43 (1.14, 1.79)	0.0021
Parkinson disease	0.99 (0.41, 2.39)	0.9856	1.06 (0.57, 1.97)	0.8583
COPD	1.85 (1.16, 2.97)	0.0100	1.59 (1.10, 2.30)	0.0140
Malignancy	4.73 (3.94, 5.68)	<0.0001	3.21 (2.83, 3.63)	<0.0001

a. Social economic status was classified as non-income (no income), low (income ranges from 1-583 US$ per month), middle (income ranges from 584-833 US$ per month) and high (income ≥ 834 US$ per month) categories with 49 patients unknown.; SLNB, sentinel lymph node biopsy; COPD, chronic obstructive pulmonary disease.
